# Development of a reference standard for the detection and quantification of *Mycobacterium avium* subsp*. paratuberculosis* by quantitative PCR

**DOI:** 10.1038/s41598-021-90789-0

**Published:** 2021-06-02

**Authors:** Monika Beinhauerova, Martina Beinhauerova, Sarah McCallum, Eric Sellal, Matteo Ricchi, Rory O’Brien, Beatrice Blanchard, Iva Slana, Vladimir Babak, Petr Kralik

**Affiliations:** 1 Department of Microbiology and Antimicrobial Resistance, Veterinary Research Institute, Brno, Czech Republic; 2 Department of Experimental Biology, Faculty of Science, Masaryk University, Brno, Czech Republic; 3 Biobest Laboratories, Milton Bridge, UK; 4 BioSellal, Dardilly, France; 5 Istituto Zooprofilattico Sperimentale della Lombardia e dell´Emilia Romagna, Piacenza, Italy; 6 Disease Research Ltd, Mosgiel, New Zealand; 7 Adiagene, Ploufragan, France; 8 Department of Animal Origin Food & Gastronomic Sciences, University of Veterinary and Pharmaceutical Sciences, Brno, Czech Republic

**Keywords:** Microbiology, Molecular biology

## Abstract

Quantitative PCR (qPCR) has become a frequently employed direct method for the detection and quantification of *Mycobacterium avium* subsp. *paratuberculosis* (MAP). The quantity of MAP determined by qPCR, however, may be affected by the type of qPCR quantification standard used (PCR product, plasmid, genomic DNA) and the way in which standard DNA quantity is determined (absorbance, fluorescence). In practice, this can be reflected in the inability to properly compare quantitative data from the same qPCR assays in different laboratories. Thus, the aim of this study was to prepare a prototype of an international MAP reference standard, which could be used to calibrate routinely used qPCR quantification standards in various laboratories to promote clinical data comparability. Considering stability, storage and shipment issues, a lyophilised fecal suspension artificially contaminated with a MAP reference strain was chosen as the most suitable form of the standard. The effect of five types of lyophilisation matrices on standard stability was monitored on 2-weeks interval basis for 4 months by F57 qPCR. The lyophilisation matrix with 10% skimmed milk provided the best recovery and stability in time and was thus selected for subsequent comparative testing of the standard involving six diagnostic and research laboratories, where DNA isolation and qPCR assay procedures were performed with the parallel use of the identical supplied genomic DNA solution. Furthermore, the effect of storage conditions on the standard stability was tested for at least 6 months. The storage at room temperature in the dark and under light, at + 4 °C, − 20 °C and − 80 °C showed no significant changes in the stability, and also no substantial changes in MAP viability were found using phage amplification assay. The prepared MAP quantification standard provided homogeneous and reproducible results demonstrating its suitability for utilisation as an international reference qPCR standard.

## Introduction

*Mycobacterium avium* subsp. *paratuberculosis* (MAP) is the causative agent of a chronic and infectious disease called paratuberculosis or Johne’s disease^1^. The disease occurs throughout the world, and the herd prevalence rates in European countries ranges from 7 to 55%^2^, although it is likely to be underestimated in many cases because of limitations of current methodologies used for detection of MAP. Paratuberculosis manifests after a long latent period lasting for years in domestic and wild ruminants and other mammals (e.g. cattle, sheep, goats, deer, wild rabbits and foxes) by a diarrhoea, wasting, emaciation, reduced milk production and ultimately ends with the death of the animal due to exhaustion. The faeces and milk of infected animals represent the major source of infection for other healthy individuals^3^. Infected milk also raises human health concerns in connection with Crohn's disease even though a direct link between MAP and disease development has not yet been established^4^. Although the pasteurization treatment of milk is commonly employed, there is an evidence demonstrating the presence of MAP in retail milk and dairy products in many parts of the world^5–7^.

The detection of MAP is traditionally based on culture on solid media, which is still widely considered as the “gold standard”. However, low sensitivity, long incubation times and sample decontamination requirements led to the development of alternative diagnostic tools, including quantitative polymerase chain reaction (qPCR)^8^. The qPCR detection and quantification are most commonly based on amplification of MAP specific targets IS900 and F57^9–11^. The IS900 sequence is present in 14–20 copies in the MAP genome and therefore is a preferable target for the sensitive detection of MAP in comparison with the single copy element F57, which in turn is preferred for MAP quantification^10^.

Using qPCR, the absolute or relative quantification of the target nucleic acid sequence in a sample may be achieved. The major advantages of absolute quantification by qPCR are, compared to relative quantification, the provision of absolute copy number of a particular target and higher reliability for comparisons. However, for the accurate determination of target quantity, properly characterized qPCR quantification standards, used for the construction of a calibration curve, are necessary^12^. The absolute quantification of targets by qPCR assay plays an important role in clinical diagnostics, food safety as well as in monitoring the transmission of diseases. Commonly used standards can take various forms, including PCR products^12^, recombinant plasmids^13,14^ or genomic DNA^15^. Variety of particular forms of the standard, methods used for its quantification as well as characteristics of the qPCR assay used in the laboratory may be reflected in demonstrable variability in qPCR results. Consequently, different protocols cannot be compared against each other^16^ resulting in differences in interpretation in disease diagnosis among laboratories, clinicians and researchers.

The determination of MAP quantity by qPCR, particularly in faeces, is essential for the correct diagnosis and classification of the infection status of animals. The reason for such precision is the phenomenon of “passive shedding” described initially by culture in heavily infected cattle herds in the USA^17^. This concept is based on findings that a high percentage of MAP shedders in infected herds shed low numbers of MAP in their faeces and after the removal of heavy shedders from the herd the overall percentage of MAP shedding animals decreases. These findings were later confirmed by qPCR analysis of the cattle faeces, which, however raised a new issue due to its higher sensitivity compared to culture^18^. Generally speaking, not all qPCR positive animals can be automatically considered to be infected and a threshold of > 10^4^ MAP cells/g of faeces has been suggested as a selection criterion between truly infected and probably “contaminated” animals^18^. Moreover, this threshold cannot be applied automatically; other factors like the size of the herd, prevalence of paratuberculosis or housing conditions must also be considered.

The passive shedding phenomenon plays therefore an important role in qPCR data interpretations and subsequent control measures adopted. Due to the differences in qPCR assays performed in different laboratories and the variety of quantification standards used, it is difficult to directly compare quantitative data from different laboratories and to find a common consensus on the determination of the threshold between infected and contaminated animals. Therefore, the aim of the study was to develop an international reference standard for quantification of MAP by qPCR, which should facilitate comparison of resulting data from different qPCR assays and from different laboratories. To achieve this goal, a suitable lyophilisation matrix was tested and chosen in the first instance. The selected matrix was then used to produce the prototype lot of the standard, which subsequently underwent interlaboratory comparison by routine DNA isolation and qPCR procedures in six diagnostic and research laboratories. Moreover, the effect of storage under various conditions on the standard stability was tested over time. This part of the study was also related to the determination of MAP cells viability in the standard within the testing period using phage amplification assay.

## Methods

### Preparation of MAP culture

**Figure 1:**
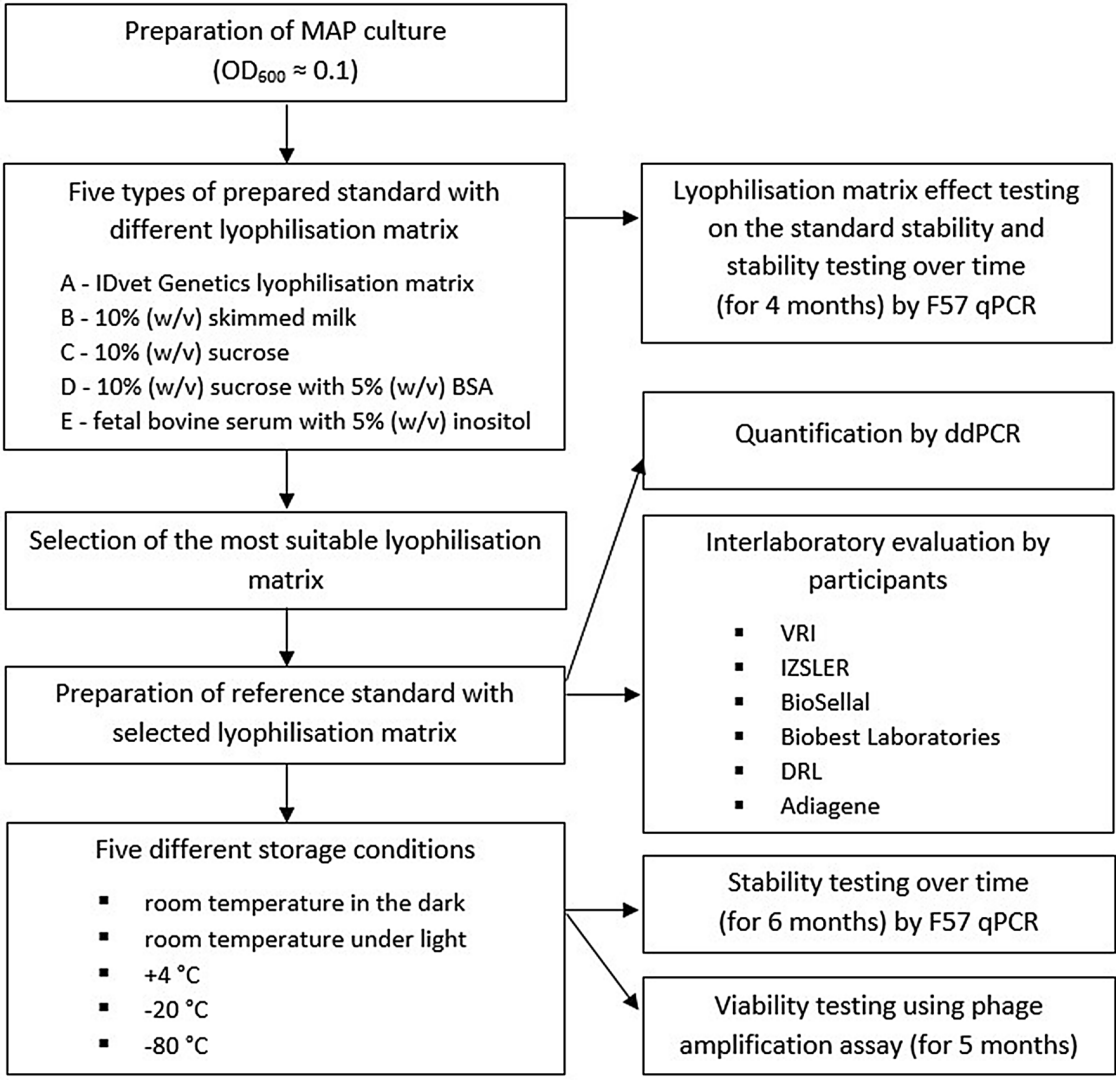
Schematic overview of the entire experimental procedure.

The field isolate of MAP (strain 7072 from a white deer, RFLP type C1) was used throughout the whole study. The isolate was grown on Herrold’s egg yolk medium (HEYM) supplemented with 2 mg/ml Mycobactin J (Allied Monitor, USA) at 37 °C for 3 weeks until visible growth was observed. Afterwards, colonies were harvested and resuspended in 1.2 ml Tris–EDTA (TE) buffer (Amresco, USA) supplemented with Carrier DNA (salmon sperm DNA, final concentration 50 ng/µl; Serva, Germany). The MAP suspension was homogenized by vortexing for 30 s following the addition of 350 mg 1 mm zirconia/silica beads (Biospec, USA). In order to remove large MAP clumps, the suspension was centrifuged at 100 g for 30 s and the upper cell fraction was resuspended in TE buffer with Carrier DNA and diluted to an optical density at 600 nm (OD_600_) of 0.1, which corresponds to approximately 10^8^ MAP cells/ml of suspension^19^. A schematic overview of the entire experimental procedure is shown in Fig. 1.

### Lyophilisation matrices—preparation of lyophilisation mixture

In total five different lyophilisation matrices were tested (Table 1) and their effect on the standard stability was monitored at 2-weeks intervals for 4 months. The lyophilisation matrices B, C and D were prepared by dissolving an appropriate amount of skimmed milk powder, sucrose (Amresco, USA) and bovine serum albumin (BSA; Sigm

**Table 1:** The lyophilisation matrices tested.

A	IDvet Genetics lyophilisation matrix^a^
B	10% (w/v) skimmed milk
C	10% (w/v) sucrose
D	10% (w/v) sucrose with 5% (w/v) bovine serum albumin
E	Fetal bovine serum with 5% (w/v) inositol

^a^Kindly provided by the Dr. Grewis and Dr. Laffont (IDvet Genetics, Montpellier, France).

a-Aldrich, USA) in sterile distilled water. The matrix E recipe was provided by The Collection of Animal Pathogenic Microorganisms (CAPM; Veterinary Research Institute, Czech Republic).

The lyophilisation mixture was composed of 40% supernatant of fecal suspension, 10% sterile water and 50% tested lyophilisation matrix. The fecal suspension was prepared from 5 g of MAP-negative bovine faeces (determined by qPCR), which were homogenized in 30 ml of sterile distilled water on a vortex and subsequently centrifuged at 100 *g* for 30 s. After mixing all three components, the lyophilisation mixture was artificially contaminated with the MAP suspension to reach an approximate amount of 10^6^ MAP cells per a vial. The lyophilisation mixture was filled into the two-ml serum glass vials (Wheaton, USA) and the lyophilisation was performed on the freeze-dryer (FreeZone Triad, Labconco, USA). The lyophilisation started with precooling to − 20 °C and continued in three steps as follows: − 20 °C for 1 h (ramp rate 5 °C/min), − 10 °C for 2 h (ramp rate 1 °C/min) and 8 °C for 5 h (ramp rate 1 °C/min). The lyophilisates were stored at room temperature in the dark.

### Determination of MAP quantity in the standard by droplet digital PCR

A total of 3 batches of lyophilised MAP standard were prepared, in which the exact number of MAP cells was independently determined using droplet digital PCR (ddPCR) targeting MAP-specific element F57. The DNA isolation was based on a slightly modified protocol from the Quick-DNA Fecal/Soil Microbe Microprep Kit (Zymo Research, USA; described in more details in chapter “DNA isolation and qPCR procedures”—*VRI*) and performed on 10 replicates. The ddPCR was carried out using QX200 droplet digital PCR system (Bio-Rad, USA) according to the manufacturer’s instructions. The assay was run in duplicate for all analysed samples in a total reaction volume of 22 μl. The reaction mixture contained 11 μl of ddPCR Supermix for Probes (Bio-Rad, USA; No UTP), 5 μl of isolated DNA and F57 probe and primers in concentrations 50 nM and 500 nM, respectively^10^. The reaction mixture was loaded with 70 μl of droplet generation oil into wells of the DG8 cartridge and placed into the QX200 droplet generator. Forty μl of generated droplets was transferred into 96-well PCR plate, which was subsequently sealed using foil heat seal and PX1 PCR plate sealer and placed in a T100 Thermal Cycler for PCR. The PCR included the following conditions: enzyme activation at 95 °C for 10 min followed by 40 cycles of a two steps thermal profile at 94 °C for 30 s and 60 °C for 60 s and a final enzyme deactivation at 98 °C for 10 min while maintaining ramp rate 2 °C/s. Following the PCR the 96-well plate was placed in the QX200 Droplet Reader, where droplets were read and analysed using QuantaSoft Software (Bio-Rad; version 1.7.4.0917). After ddPCR analysis the MAP quantity determined was recalculated per a vial according to the appropriate DNA isolation procedure parameters and this value was considered as a reference.

### Stability of MAP reference standard over time and effect of lyophilisation matrices on standard stability

The DNA isolation from a lyophilised MAP reference standard, stored at room temperature in the dark, was based on a slightly modified QIAamp DNA Stool Mini Kit protocol (Qiagen, Germany)^19^ including mechanical homogenisation in a bead beating instrument (MagNa Lyser) and elution to 100 µl of TE buffer. The isolated DNA was used for the quantification of MAP cells by a qPCR assay amplifying the single copy fragment F57^10^ on a LightCycler 480 instrument (Roche Molecular Diagnostic, Germany). Each sample was analysed in technical duplicates. The absolute numbers of MAP cells were determined using ‘Fit-point analysis’ in LightCycler 480 software (version 1.5.1.62) according to a calibration curve derived from the serial dilution of circular plasmid standard containing the F57 insert.

The lyophilisation matrix effect testing and initial determination of repeatability and stability in time of the MAP reference standard were performed. After the lyophilisation (day 1), eight vials from each type of the lyophilisation matrix underwent to the DNA isolation and F57 qPCR to assess the quantity of MAP cells and repeatability. To test the stability, two vials of each type of the lyophilisation matrix were analysed at fortnightly intervals for 4 months.

### Interlaboratory testing of MAP reference standard

In order to evaluate the MAP reference standard performance in different laboratories, the prototype lot of the standard was prepared and 10 vials of lyophilised standard (with 10% skimmed milk solution as a lyophilisation matrix, which has shown the best performance in previous experiment) were sent out to six laboratories: Veterinary Research Institute (VRI, Czech Republic), Istituto Zooprofilattico Sperimentale della Lombardia e dell'Emilia Romagna (IZSLER, Italy), BioSellal (France), Biobest Laboratories (UK), Disease Research Limited (DRL, New Zealand) and Adiagene (France). Each participating laboratory performed its own routine DNA isolation using one or more DNA isolation kit and one or more PCR assays as described below. To compare resulting data from different laboratories, the lyophilised MAP genomic DNA (gDNA) originating from the MAP strain 7072 was included in testing together with standards. The laboratories were instructed to dilute the gDNA and construct an external independent calibration curve for quantification of the MAP DNA in each qPCR run.

#### Preparation of MAP gDNA standard

MAP gDNA standard was isolated by a modified protocol from DNeasy Blood and Tissue Kit (Qiagen). Briefly, colonies of MAP were harvested and resuspended in 360 µl of enzymatic lysis buffer, prepared according to the manufacturer’s instructions, followed by incubation at 37 °C with shaking at 1400 rpm for 30 min. Subsequent purification steps followed the manufacturer's instructions. The concentration of isolated DNA was measured by NanoDrop 2000c (Thermo Scientific, USA). The DNA (100 ng) was pipetted into screw cap tubes and dried on a thermoblock (Major Science, USA) for about 3 h.

The dried MAP gDNA was reconstituted in 100 µl of water (LightCycler 480 Probes Master H_2_O, PCR grade, Roche Molecular Diagnostics) to a concentration of 1 ng/µl, which was determined by ddPCR to correspond to 2 × 10^5^ MAP cells/µl. The reconstituted gDNA was diluted tenfold in a range from 2 × 10^5^ to 2 × 10^–1^ cells/µl (concentration range 1 ng/µl–1 fg/µl) and amplified in technical duplicate in parallel with samples and standards in all qPCR assays performed.

#### DNA isolation and qPCR procedures

I. VRI
  DNA from lyophilised MAP reference standards was isolated by a slightly modified protocol from the Quick-DNA Fecal/Soil Microbe Microprep Kit (Zymo Research). The lyophilisates were dissolved in 750 µl of BashingBead Buffer and transferred to a BashingBead Lysis Tube. Then the samples were subjected to mechanical homogenisation in the MagNA Lyser at 6400 rpm for 30 s, left until they have cooled down and the homogenization step was repeated. Following centrifugation at 10,000*g* for 1 min, 400 µl of supernatant was processed using an isolation kit according to the manufacturer's instructions. The DNA was eluted into 25 µl of DNA Elution Buffer and 5 µl was used as a template for the F57 qPCR assay as described above^10^.
II. IZSLER
  The lyophilisates were dissolved in 500 µl of DEPC (diethyl pyrocarbonate) water and submitted to mechanical homogenisation in Tissue Lyser II (Qiagen) at 30 Hz for 10 min with 10 mg of acid washed glass beads (Sigma-Aldrich, Milan, Italy). Two-hundred µl of the supernatant were processed using DNeasy Blood & Tissue Kit (Qiagen) by adding 200 µl of buffer AL, 20 µl of Proteinase K and incubating for 10 min at 70 °C. After addition of 200 µl of ethanol, the mixture was centrifuged through columns provided by the kit. After washing according to the manufacturer's instructions, the DNA was eluted into 200 µl of DNA Elution Buffer and 5 µl were used in IS900 qPCR assay already used^20^ and carried out on a StepOne qPCR system (Thermo Fisher, software version 2.3).
III. BioSellal
  The lyophilisates were resuspended in 600 µl of ATL buffer (Qiagen) and transferred to a grinding tube (BioPrep MAP-2). The tubes were grinded in a FastPrep^®^ grinder (MP BioMedicals) 4 times for 45 s at 6.5 m/s. After centrifugation at 10,000*g* for 1 min, 400 µl of supernatant was used for the DNA extraction using BioExtract^®^ Superball^®^ and BioExtract^®^ Column (BioSellal) according to the manufacturer’s instructions. Five lyophilisates were extracted per extraction methods. The elution volume was 100 µl for both extraction kits. The detection and quantification of the MAP IS900 sequence was performed, according to the manufacturer’s instructions, using 5 µl of extracted DNA and Bio-T kit^®^ Mycobacterium avium paratuberculosis (BioSellal) on AriaMx thermal cycler (Agilent Technologies).
IV. Biobest Laboratories
  In the extraction protocol 1, DNA from lyophilised MAP standard material was extracted by a slightly modified protocol from the MagMAX Total Nucleic Acid kit (Applied Biosystems, USA). The lyophilised material was reconstituted with 300 µl MagMAX lysis/binding solution, this material was then transferred to a bead beating tube and processed as per the manufacturer’s guidelines. In the extraction protocol 2, DNA from lyophilised MAP standard material was extracted by a slightly modified protocol from the MagMAX CORE kit with mechanical lysis module (Applied Biosystems). The lyophilised material was reconstituted with 400 µl MagMAX CORE clarifying solution, this material was then transferred to a bead beating tube and processed as per the manufacturer’s guidelines. Five vials of lyophilised MAP standard material were processed by each extraction method.
  DNA from each extract was tested in duplicate by two PCR tests. PCR 1 used the VetMAX-Gold MAP PCR kit (Applied Biosystems) with an additional 1.3 µl of water, 1.5 µl of IS900 primers and 1.2 µl of IS900 probe (primers and probes are described in study by Ravva and Stanker^21^). PCR 2 also used the VetMAX-Gold MAP PCR kit (Applied Biosystems) with an additional 4 µl of water. In both PCR reactions, 4 µl of DNA was added to a total mastermix of 21 µl. PCR tests were run on the Rotor-Gene 6000 (previously Corbett Life Science, now sold by Qiagen as the Rotor-Gene Q).
V. DRL
  DNA from the lyophilised MAP reference standards was recovered using either of two different methods representing protocols employed routinely by the DRL laboratory for low-throughput (n ≤ 5) or high-throughput sample numbers. For low-throughput extractions, individual spin columns (Quick-DNA Fecal/Soil Microbe Microprep Kit; Zymo Research) were utilised with minor modifications to the supplied protocol. Briefly, the reference standards were resuspended in 1 ml of the kit lysis buffer and transferred to a 2 ml skirted, screw top microcentrifuge tube containing 0.5 ml of a 50:50 mix of 0.5 and 0.1 mm zirconia beads (BioSpec, Corp., Bartesville, OK, USA). Samples were mixed briefly before the tube was placed in a vigorously boiling water bath for 5 min. Following boiling lysis, samples were immediately further disrupted by bead beating for 5 min at 1750 bpm in a GenoGrinder instrument (Spex SamplePrep, NJ, USA). Following bead beating, the samples were centrifuged at 18,000*g* for 5 min to pellet undissolved solids before 370 µl of cleared lysate was removed and processed for DNA extraction according to the manufacturer’s protocol. Nucleic acids were eluted in 110 µl of DNA elution buffer and 3 µl was used for PCR amplification.
  For high-throughput extractions, a 96 well plate extraction format was utilised. Samples were resuspended in 1100 µl of lysis buffer and subjected to thermal, chemical and mechanical lysis. Eight hundred microliters of clarified lysate were extracted on a semiautomated DNA extraction platform resulting in 150 µl of eluted DNA. Three microliters of eluate were used for PCR amplification.
  For qPCR quantitation, samples were assayed for IS900 using an in-house, hydrolysis probe based real time PCR assay using an Applied Biosystems 7500 Fast Real-Time PCR System (Life Technologies, Carlsbad, CA, USA).
VI. Adiagene
  Two DNA isolation procedures were applied to the lyophilised MAP reference standards. In the isolation procedure 1, five lyophilisates were dissolved in 500 µl of Nuclease free water and transferred to a tube containing 300 mg glass beads (ADIAPURE™ aliquoted Glass beads, Bio-X Diagnostics, Belgium). Samples were grinded in the Mixer Mill at 30 Hz for 10 min and centrifuged at 15,000*g* for 5 min. 350 µl of supernatant was used for DNA extraction using QIAamp DNA Mini kit (Qiagen).
  In the isolation procedure 2, five lyophilisates vials were dissolved in 400 µl of ATL buffer and proteinase K from QIAamp DNA Mini kit. Samples were incubated for 1 h at 56 °C and then transferred to a tube containing 300 mg glass beads (ADIAPURE™ aliquoted Glass beads). Samples were grinded in the Mixer Mill at 30 Hz for 10 min and centrifuged at 15,000*g* for 5 min. Subsequently, 220 µl of supernatant was used for DNA extraction using QIAamp DNA Mini kit.
  All DNA samples were eluted with 200 µl of AE buffer and 5 µl was used as a template for the qPCR assay using ADIAVET™ PARATB REAL TIME according to the manufacturer’s instructions (Bio-X Diagnostics).

### The effect of storage conditions on the standard stability over time and determination of MAP cells viability using phage amplification assay

To evaluate the MAP reference standard stability under different storage conditions, the vials with lyophilised standards were stored under five conditions: room temperature in the dark, room temperature under light, + 4 °C, − 20 °C, − 80 °C and all were analysed in 2-weeks intervals for at least 6 months. DNA from the lyophilised standards was isolated using Quick-DNA Fecal/Soil Microbe Microprep Kit as described above with analysis by F57 qPCR assay^10^. DNA isolation was carried out in duplicate from each condition tested and the gDNA standard gradient (described above) was included in each qPCR run performed and used for quantification.

In parallel with the DNA isolation and F57 qPCR, the slightly modified phage amplification assay previously described by Stanley et al.^22^ was performed. The goal was to assess stability of the MAP cells in the standard in a complex manner. Specifically, two vials of lyophilised standard from each storage condition were analysed in 2-weeks intervals. The vials were dissolved in 1 ml of Middlebrook 7H9 broth (Difco Laboratories, USA) supplemented with 10% Middlebrook OADC enrichment (Becton Dickinson, USA), 2 mM CaCl_2_ (Penta, Czech Republic) and 1.25% PANTA (Becton Dickinson). The antibiotic mixture PANTA was prepared according to the manufacturer's instructions and was used to suppress natural microflora present in faeces. After overnight incubation at 37 °C with shaking at 100 rpm, 100 µl of bacteriophage D29 (kindly provided by Dr. Botsaris, Cyprus University of Technology, Cyprus and Dr. Rees, University of Nottingham, UK; stock concentration 10^9^ PFU/ml) was added to each sample and incubated at 37 °C for 2 h with shaking at 100 rpm. Then the bacteriophages that have not infected mycobacterial cells were eliminated by the addition of 100 µl of 100 mM ferrous ammonium sulphate (FAS; Lach-Ner, Czech Republic). After incubation of samples at room temperature for 5 min, 5 ml of enriched medium was added for FAS neutralization followed by the preparation of tenfold dilutions of the samples. Then 1 ml of *Mycobacterium smegmatis* mc^2^155, grown for 48 h at 37 °C with shaking at 100 rpm in 50 ml of Middlebrook 7H9 broth with 10% OADC (10^8^ CFU/ml), was added to each sample dilution. All samples were transferred into a sterile Petri dish and 6 ml of molten 1.6% Middlebrook 7H10 agar (Difco Laboratories) cooled to 55 °C was added to each plate. The content of the plate was carefully mixed and left at room temperature until the agar set. The number of plaques formed was counted after overnight incubation of plates at 37 °C. Positive and negative controls were included in each analysis.

### Mathematical and statistical analysis

#### Stability of MAP reference standard over time and effect of lyophilisation matrices on standard stability

The repeatability and stability over time of standards with a different lyophilisation matrix (A–E) was tested using one-way analysis of variance (ANOVA). The repeatability and stability were estimated from the model as "mean square error of sample (within-subject variance)" and “mean square error of Week (within-subject variance)”, respectively. In addition, R^2^, mean, sample standard deviation and coefficient of variation were calculated.

#### Interlaboratory testing of MAP reference standard

The MAP DNA copy number detected by a particular isolation procedure and qPCR assay was calculated from the raw crossing point (Cq) values according to the respective regression equation of gDNA gradient from the actual run. This value was then converted to the amount of MAP cells in the initial sample (lyophilised vial) according to the appropriate isolation procedure parameters using this formula:

$$y= copy \; number \times \frac{elution \; volume}{volume \; of \; DNA \; in \; qPCR \; reaction} \times \frac{volume \; to \; dissolve \; the \; lyophilisate}{volume \; of \; lysate \; collected \; for \; subsequent \; isolation \; steps}$$

Particular recalculation formulas for individual DNA isolation and PCR procedures used by participating laboratories are given in Table 2. The mean with standard deviation for the particular DNA isolation and qPCR assay performed were calculated. For statistical evaluation of mean MAP quantity estimations in the standard obtained by each method in the participating laboratory, one-way ANOVA and one-sample t-test were used.

**Table 2:** The number of MAP cells in the lyophilised standard detected by various DNA isolation procedures and PCR assays in different laboratories.

Laboratory	Method	Recalculation^c^	Mean of detected cells	SD
VRI^a^		GE number^d^ × (25/5) × (750/400)	1.82 × 10^6^	3.63 × 10^5^
IZSLER^a^		GE number × (200/5) × (500/200)	5.16 × 10^5^	2.07 × 10^5^
BioSellal	BioExtract^®^ Column^b^	GE number × (100/5) × (600/400)	9.75 × 10^5^	1.33 × 10^5^
	BioExtract^®^ Superball^®^^b^	GE number × (100/5) × (600/400)	4.56 × 10^5^	8.40 × 10^4^
Biobest Laboratories	**Isolation method 1**			
	PCR 1^b^	GE number × (50/4) × (300/200)	3.20 × 10^5^	5.28 × 10^4^
	PCR 2^b^	GE number × (50/4) × (300/200)	3.08 × 10^5^	5.04 × 10^4^
	**Isolation method 2**			
	PCR 1^b^	GE number × (90/4) × (400/300)	6.40 × 10^5^	2.35 × 10^5^
	PCR 2^b^	GE number × (90/4) × (400/300)	6.23 × 10^5^	2.28 × 10^5^
DRL	Low throughput extractions^b^	GE number × (110/3) × (1000/370)	2.84 × 10^5^	1.68 × 10^4^
	High throughput extractions^b^	GE number × (150/3) × (1100/800)	1.42 × 10^6^	1.23 × 10^5^
Adiagene	Isolation method 1^b^	GE number × (200/5) × (500/350)	1.02 × 10^6^	4.62 × 10^5^
	Isolation method 2^b^	GE number × (200/5) × (400/220)	7.34 × 10^5^	3.10 × 10^5^

*GE* genome equivalent, *SD* standard deviation.

^a^Data originated from 10 repeats.

^b^Data originated from 5 repeats.

^c^Recalculation of MAP quantities per a vial.

^d^Number of MAP genome equivalents detected by qPCR.

#### The effect of storage conditions on standard stability over time and determination of MAP cells viability using phage amplification assay

The stability and cell viability of MAP standard stored under five conditions over time were analysed using two-way ANOVA.

## Results

### Preparation of MAP reference standard

With regard to stability, storage and shipment issues, the lyophilised form of the MAP reference standard appeared to be the most suitable. The optimised protocol for a large scale (approximately 200 vials) lyophilisation provided the successful freeze drying of all five standard types with a different lyophilisation matrix. A highly porous and easy to reconstitute cake was obtained for all types of lyophilised standard.

In the 1st batch of lyophilised standard, intended for the interlaboratory testing, the exact MAP quantity was determined to be 1.0 × 10^6^ cells. In the 2nd and 3rd batch, which were used for the stability study under five storage conditions, the MAP quantities were defined to be 9.3 × 10^5^ and 9.8 × 10^5^ cells, respectively. Only for the initial stability study evaluating various lyophilisation matrices, the exact MAP quantity determination in lyophilised vials was not performed.

### Stability of MAP reference standard over time and effect of lyophilisation matrices on standard stability

**Figure 2:**
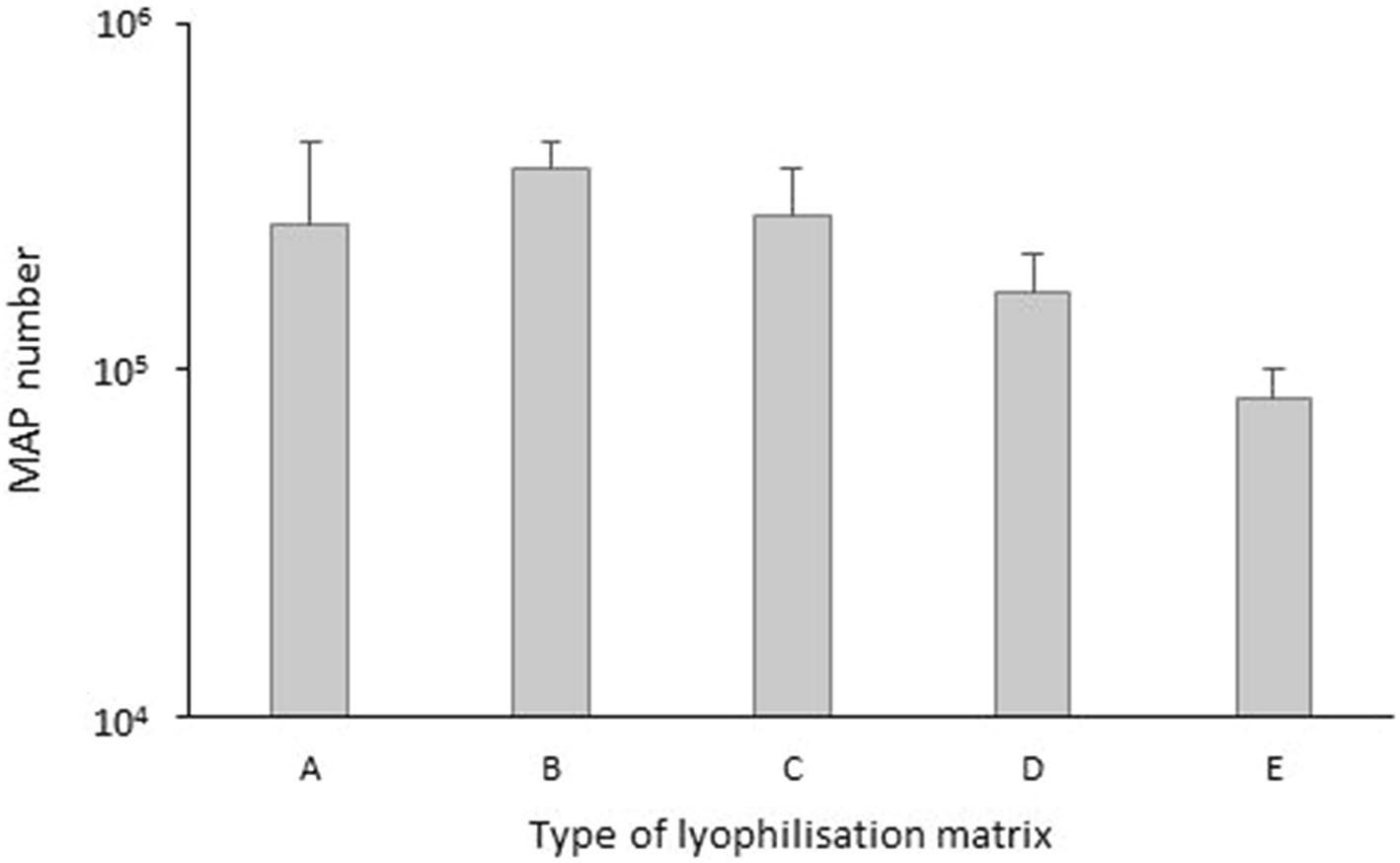
The mean amount of MAP cells recovered using F57 qPCR in day 1 from five types of standard with different lyophilisation matrix. Error bars represent standard deviations obtained from eight biological replicates.

**Figure 3:**
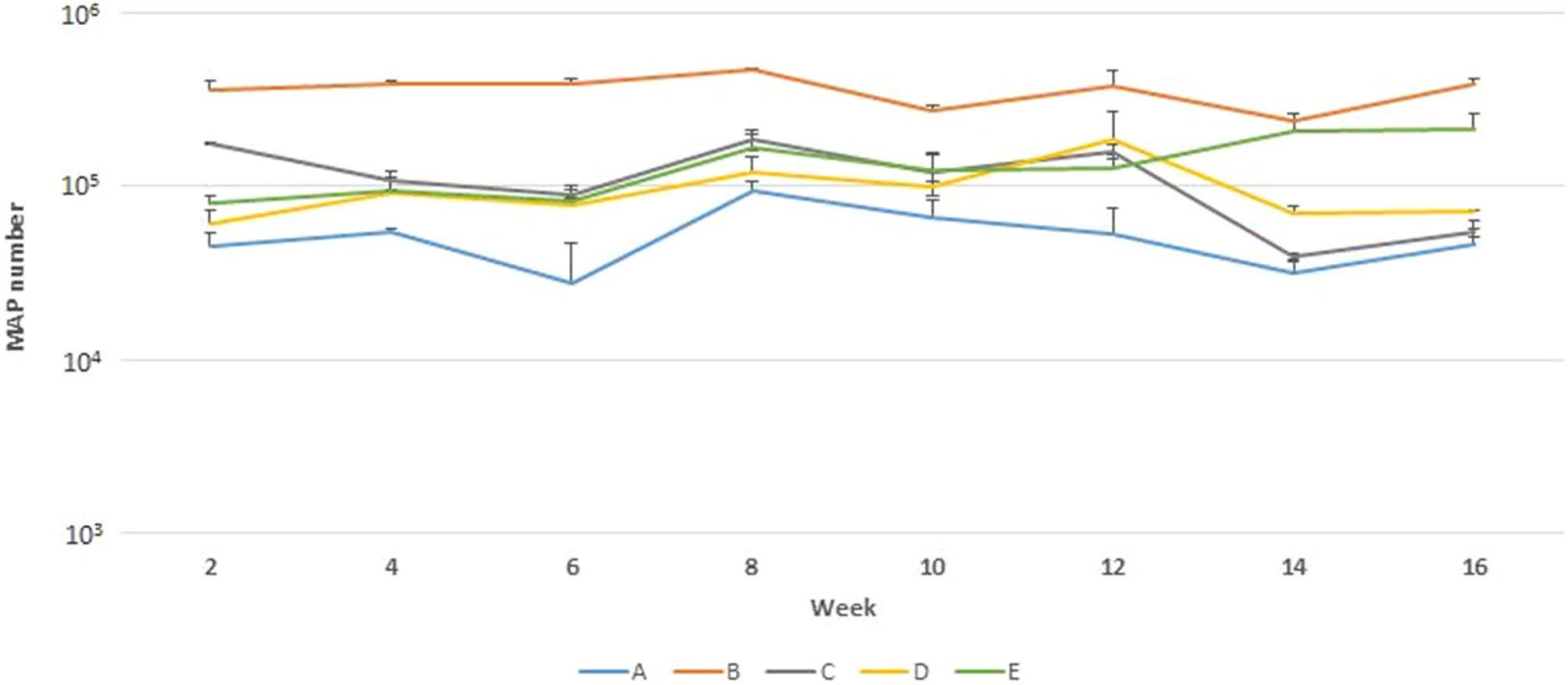
The stability of MAP reference standard with five different lyophilisation matrices in time determined by F57 qPCR assay. Error bars represent standard deviations for the means of two biological replicates.

The determination of MAP cells quantity in eight biological replicates of the standard by F57 qPCR assay analysed immediately after the lyophilisation demonstrated a high repeatability of four types (B–E) of standard tested (Fig. 2; Table 3). The best uniformity of results and the highest yield were recorded for the standard with lyophilisation matrix B (10% skimmed milk solution) during the 16 weeks of the initial stability determination (Fig. 3; Table 4). Based on these parameters, the 10% skimmed milk solution was selected as the most suitable lyophilisation matrix for the preparation of the lyophilised reference standard. The selected lyophilisation matrix was used in further testing.

**Table 3:** Descriptive statistic regarding repeatability testing of five types of standard with different lyophilisation matrix.

	A	B	C	D	E
Repeatability	0.346	0.012	0.058	0.044	0.015
R^2^ of model	0.998	0.885	0.936	0.982	0.933
Mean	5.271	5.576	5.418	5.205	4.914
SD	0.402	0.081	0.171	0.145	0.087
Coeff. of variation (%)	7.63	1.45	3.15	2.79	1.77

**Table 4:** Descriptive statistic regarding stability testing of five types of standard with different lyophilisation matrix.

	A	B	C	D	E
Variance	0.153	0.036	0.183	0.079	0.117
R^2^ of model	0.556	0.763	0.929	0.641	0.724
Mean	4.662	5.542	5.036	4.942	5.094
SD	0.249	0.104	0.218	0.170	0.191
Coeff. of variation (%)	5.35	1.87	4.32	3.43	3.75

### Interlaboratory testing of MAP reference standard

The performance of the lyophilised MAP reference standard was evaluated in six diagnostic and research laboratories by various isolation and qPCR methodologies routinely employed by these laboratories. The number of MAP cells detected by qPCR were recalculated per a vial according to the parameters of the particular DNA isolation (Table 2). Comparability of the resulting data from different laboratories was ensured by the utilization of MAP gDNA, the exact concentration of which was determined using ddPCR and which was used as a calibrator for quantification of the analysed standards by each laboratory. The statistically significant variability in MAP quantity estimates obtained by different laboratories and methods used was recorded (P < 0.01; ANOVA factor effect F-test) with estimations ranging from 2.84 × 10^5^ to 1.82 × 10^6^.

### The effect of storage conditions on standard stability over time

**Figure 4:**
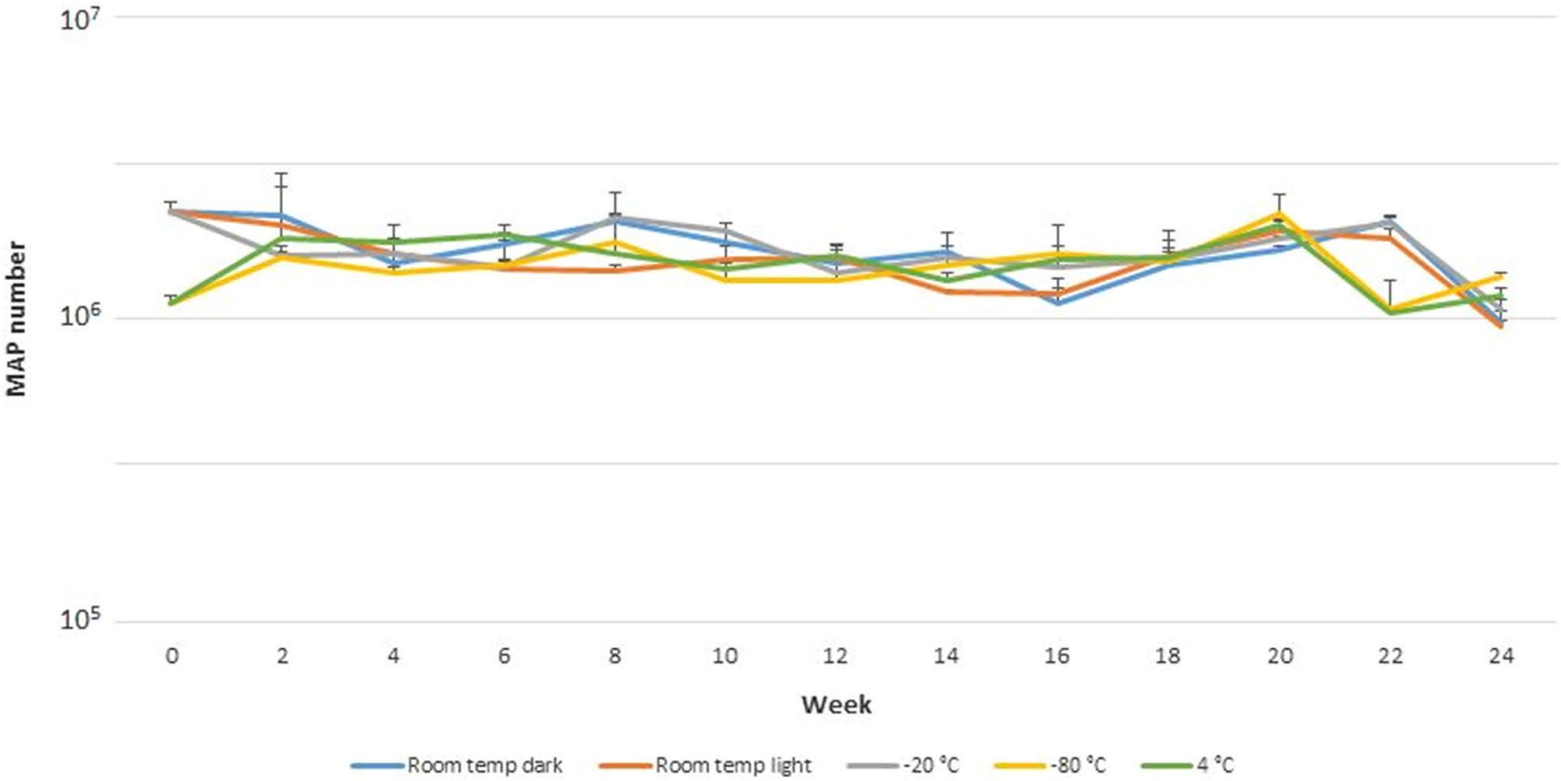
The stability of MAP reference standard stored in five different storage conditions over time determined by F57 qPCR assay. Error bars represent standard deviations for the means of two biological replicates.

No statistically significant differences were found between the individual storage conditions (P > 0.05; ANOVA factor effect F-test; Fig. 4) during 6-month testing period, therefore, all five storage conditions can be considered equivalent in terms of the effect on standard stability. Also, differences in MAP quantity estimates in lyophilised standard obtained during the testing period are not statistically significant (P > 0.05; ANOVA factor effect F-test).

### Determination of MAP cells viability using phage amplification assay

**Figure 5:**
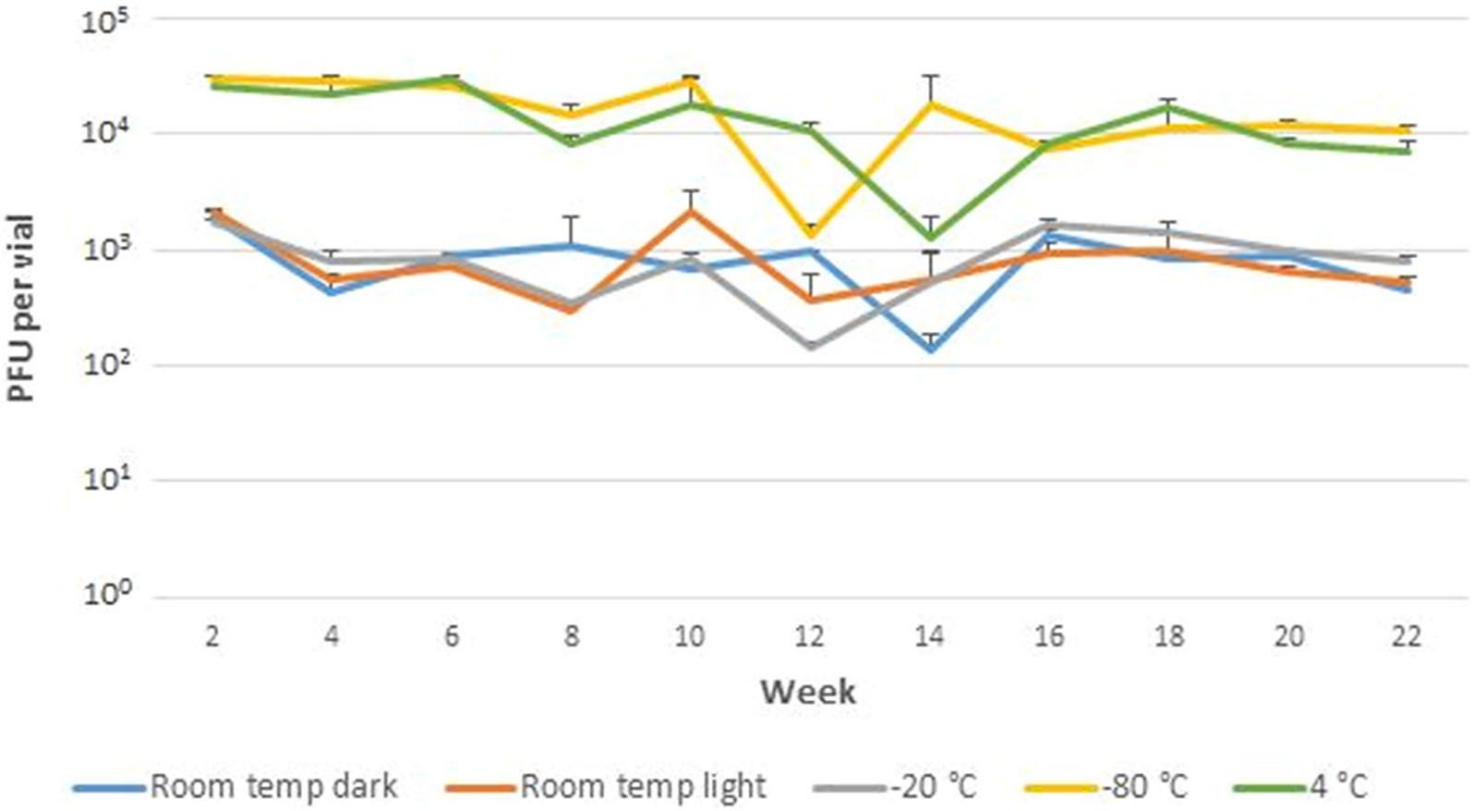
The viability of MAP cells in the reference standard stored in five storage conditions over time determined by phage amplification assay. Error bars represent standard deviations for the means of two biological replicates.

The difference in viable MAP quantities determined for the standard stored at room temperature in the dark, under the light and at − 20 °C and for the standard stored at − 80 °C and + 4 °C was recorded (Fig. 5). This can be explained by separate preparations of these standards due to the high demand for a number of lyophilised vials for the stability study. However, no substantial difference in trends during 5-months testing period was observed between the two batches. The MAP viability has been relatively constant over time under all five storage conditions. The main deviation was the decrease in the values of viable MAP cells of around one order of magnitude in the 12th and 14th week.

## Discussion

The aim of the present study was to develop an international reference standard for accurate MAP quantification by qPCR. Nowadays, no reference standard for MAP quantity determination is available; various diagnostic and research laboratories use different DNA extraction and subsequent amplification procedures, whether commercial or in-house. Utilization of variety of calibration materials (recombinant plasmid, genomic DNA, PCR product, etc.) and methods for DNA quantity assessment of these materials significantly influences the results of particular qPCR and subsequent data interpretation^12,14,15^. Besides, the degradation of calibration materials maintained in a frozen state over time can significantly affect PCR efficiency^12^. These combined sources of variability result in the inability to compare quantitative data from various qPCR assays from different laboratories^23^. Through the establishment of a reference standard, it is possible to address this issue and promote unification of DNA quantification standards (independently on the DNA extraction and qPCR) in qPCR detection and quantification of pathogens. Universally available reference materials for nucleic acid quantity testing have been established, for example, for hepatitis viruses, human immunodeficiency virus and human parvovirus B19^24–28^, which play an important role in blood screening and diagnostic fields. The consensus in quantitative DNA results between clinical laboratories and between serial samples from a particular patient within one laboratory is essential for definition of generally accepted clinical threshold values for viral infection and for monitoring disease initiation and progression^16^.

In diagnosis of paratuberculosis, qualitative interpretation of qPCR data is not sufficient for the determination of infectious status of animals due to the effect of passive shedding phenomenon. The passive shedding where MAP is present in animal faeces as a result of the oral ingestion of MAP organisms from environment massively contaminated by MAP but unrelated to the current host infection with paratuberculosis, was first demonstrated by fecal culture and later confirmed by qPCR testing of fecal samples^18,29^. As molecular methods such as qPCR are capable of detecting very low levels of DNA, establishment of cut-off value is relevant in order to correctly conclude whether the presence of MAP in faeces is the result of real ongoing infection of animals or only reflects a high environmental infectious pressure. Thus, the quantity of 10^4^ MAP cells/g of faeces has been suggested as the threshold to discriminate between truly infected and probably “contaminated” animals^18^. The accurate determination of MAP load in faeces and correct interpretation of qPCR data are crucial for the reliable animal infection status assessment and subsequent reasonable paratuberculosis control measures adopted. The variability in the efficiency of DNA extraction from fecal sample and amplification by the particular qPCR may lead to the underestimation of MAP load in the clinical sample, which can affect subsequent decision regarding control or eradication interventions and affect the prevalence of MAP-infected individuals in the herd.

The lyophilised form of the MAP reference standard, where residual moisture and oxygen are minimized, was decided to be the most suitable as this form can be dispatched at ambient temperature and is stable over time^30^. The composition of the standard is based on a fecal suspension, which is also of advantage as it closely simulates a real sample. In total, five types of lyophilisation matrix were analysed, and after freeze-drying there were no visible significant differences among lyophilisation matrices used. Also, all five types were easily reconstituted in a lysis buffer. The lyophilised standard with 10% skimmed milk as a lyophilisation matrix (type B), however, provided the best recovery and stability over time and was thus subjected to further testing.

To ensure accurate and independent MAP quantity value determination in the MAP reference standard, the ddPCR was utilized and shown to be the most appropriate approach. The ddPCR, a single-molecule amplification technique based on limiting dilution, is a highly accurate and precise method for absolute DNA quantification, which does not require the creation of the calibration curve and exhibits increased tolerance to inhibitors compared to qPCR. Currently, the ddPCR is considered as a higher-order reference measurement method for nucleic acid testing of infectious disease^31,32^. In present study, three batches of lyophilised MAP standard were separately prepared and independently quantified using ddPCR with all results oscillating around the theoretical value of 10^6^ MAP cells per a vial (derived from the rough OD determination), which demonstrates ddPCR to be a robust and reliable technique for exact reference standard quantification. These values corresponded to the amount of 10^6^ MAP cells from the subsequent qPCR analyses. In some cases, however, minimal deviations were recorded between qPCR results and the MAP quantity in the standard determined by ddPCR, which can be attributed to manipulation with samples including dilutions and pipetting errors. The ddPCR procedure used here have proven its suitability in an earlier study when allowed to achieve a defined quantity of quantification plasmid standards for various viral and bacterial pathogens^33^. Besides, the ddPCR technology was reported to produce more precise, reproducible and statistically significant results required for publication quality data for samples with low levels of nucleic acids and/or variable amounts of chemical and protein contaminants^34^.

No significant effect on MAP standard stability was found over 6 months under five different storage conditions using F57 qPCR, keeping the number of MAP cells recovered from the standard at a roughly constant level during testing period. These findings were supported by results of a phage amplification assay, the method allowing a rapid enumeration of viable MAP cells in various kinds of matrices including milk and faeces using a lytic mycobacteriophage D29^35^. No substantial changes in number of viable MAP cells in the standard were found during 5 months of testing period. The main deviation recorded was the decrease in the values of viable MAP cells of around one order of magnitude in the 12th and 14th week, however, this decrease occurred in all storage conditions and may therefore be due to some circumstances other than properties of the samples. It is likely that this was due to a combination of various factors, including pipetting, sample manipulations, and the possible presence of MAP cells clumps in the sample may also play a role. Two batches of lyophilised standard were prepared for stability study under five storage conditions, which was reflected in the number of viable MAP cells determined. In one batch (room temperature in the dark and under light, and − 20 °C) the value of viable MAP cells was of the order of 10^3^, while for the next batch (− 80 °C and + 4 °C) this value was around 10^4^ cells. Nevertheless, the determined viability did not affect the qPCR results. The viability determination showed that the standard in its current form is rather not suitable for the culture standardization.

Although the values of viable MAP cells in the standard determined by phage amplification assay were up to about three orders of magnitude lower than qPCR values, this is in concordance with results of cultivation experiments of Kralik et al.^19^ when cultivation provided lower MAP CFU counts by approximately two log_10_, compared to F57 qPCR. This was explained by the tendency of MAP cells to form clumps and the ability of cultivation to detect only viable cells. The presence of fecal suspension in a standard, that has been found to contain components inhibiting phage infection^35^, may also contribute to a further reduction in number of cells detected by the phage amplification assay. Moreover, for phage amplification assay, the antibiotic mixture PANTA was necessary to be added to the media to suppress the growth of natural microflora present in the fecal suspension. The microflora not only competed with the growth of "background" formed by *M. smegmatis* cells, but also hampered the counting of plaques on an agar. Overall, the results of the stability study show that a short time at elevated or decreased temperatures during shipment should not affect MAP quantity or viability of the standard.

To demonstrate the routine application of the standard and to evaluate the MAP reference standard performance in various laboratories from different countries, the interlaboratory study was performed. Certain differences (< 1 log_10_) were found among MAP quantity estimations, which reflects various methodologies (particularly efficiencies of the DNA isolation procedures) that were employed by participants. However, these differences in quantitative data can be considered as minor in comparison with previous studies performed to establish other international reference standards for hepatitis viruses and human parvovirus B19^26,28,36^ that reached 1.5–2.5 log_10_ difference. In addition, with the exception of four results, all fell within acceptable standards of variation, defined as the expected result ± 0.50 log_10_^37^. On the other hand, the achieved results variance underlines the importance of introducing a reference standard for accurate and uniform MAP quantity value determination by qPCR assays.

The intended concept of using MAP reference standard considers calibration of the quantification standard of a particular qPCR according to the MAP reference standard. If the uniform procedure would be used for extraction and quantification of DNA from both the reference standard and fecal samples, the DNA losses during the DNA extraction and amplification process of samples would be eliminated enabling measurement precision of a MAP load in the sample. In addition to that, quantities of DNA in samples determined in different laboratories based on this reference standard could therefore be more comparable and interpretation criteria could be unified.

This study was performed with the aim of establishing an international reference standard for the exact MAP quantification using qPCR and to facilitate comparability of quantitative data among various laboratories. Obtained results demonstrated striking data homogeneity of prepared MAP reference standard. The applicability of the reference standard for routine MAP quantity assessment by qPCR was proven through interlaboratory testing involving six diagnostic and research laboratories employing different PCR assays and through stability study over time in various storage conditions. The introduction of the first international reference standard for MAP qPCR assay calibration would be an initial important step in quality improvement and standardization of this laboratory tool. The next step will be an applying for approval from the OIE Biological Standards Commission to include qPCR standard among OIE-Approved Reference Standards.
